# Optical, Structural, and Biological Characteristics of Rapid-Sintered Multichromatic Zirconia

**DOI:** 10.3390/biomedicines13102361

**Published:** 2025-09-26

**Authors:** Minja Miličić Lazić, Nataša Jović Orsini, Miloš Lazarević, Vukoman Jokanović, Vanja Marjanović, Branimir N. Grgur

**Affiliations:** 1 Faculty of Dentistry, University of Belgrade, 11000 Belgrade, Serbia; milos.lazarevic@stomf.bg.ac.rs (M.L.); vanja.marjanovic1.9.9@gmail.com (V.M.); 2 Vinča Institute of Nuclear Sciences—National Institute of the Republic of Serbia, University of Belgrade, 11001 Belgrade, Serbia; natasaj@vin.bg.ac.rs (N.J.O.); vukoman@vinca.rs (V.J.); 3 Faculty of Technology and Metallurgy, University of Belgrade, 11120 Belgrade, Serbia; bngrgur@tmf.bg.ac.rs

**Keywords:** biomaterials, multichromatic zirconia, monolithic crown, cell proliferation, cell adhesion, fibroblasts, CIELAB, color space, XRD analysis

## Abstract

**Background:** To overcome the esthetic limitations of dental monolithic zirconia restorations, multichromatic systems were developed to combine improved structural integrity with a natural shade gradient that mimics the optical properties of natural teeth. In response to the clinical demand for time-efficient, i.e., chairside fabrication of zirconia restorations, rapid sintering protocols have become necessary to adjust clinical efficiency along with material performance. This study addresses the challenges of a rapid sintering protocol related to optical performance and phase transformation of the final restoration and the zirconia–cell interaction. **Methods:** The influence of a rapid sintering protocol on the color stability of the final dental restoration was evaluated by the CIE L*a*b* color space. Phase transformation was assessed through X-ray diffraction analysis. Cellular behavior was evaluated by measuring wettability, the material’s surface energy, and a cell mitochondrial activity assay on human gingival fibroblasts. **Results:** Optical measurements demonstrated that the total color change in all layers after rapid sintering was above the perceptibility threshold (ΔE* > 1.2), while only the polished enamel layer (ΔE* = 3.01) exceeded the acceptability threshold (ΔE* > 2.7), resulting in a clinically perceptible mismatch. Results of X-ray diffraction analysis, performed for fixed occupancy at Z_0.935_Y_0.065_O_0.984_, revealed that rapid sintering caused a decrease in the cubic (C-) phase and an increase in the total amount of tetragonal (T-) phases. Conventionally sintered zirconia consists of 54% tetragonal (T-) and 46% cubic (C-) phase, whereas in the speed-sintered specimens, an additional T1 phase was detected (T = 49%; T1 = 27%), along with a reduced cubic fraction (C = 24%). Additionally, a small amount of the monoclinic (M) phase is noticed. Although glazing as a surface finishing procedure resulted in increased hydrophilicity, both polished and glazed surface-treated specimens showed statistically comparable cell adhesion and proliferation (*p* > 0.05). **Conclusions:** Rapid sintering induced perceptible color changes only in the enamel layer of multichromatic zirconia, suggesting that even layer-specific alterations may have an impact on the overall esthetic outcome of the final prosthetic restoration. Five times higher heating and cooling rates caused difficulty in reaching equilibrium, leading to changes in lattice parameters and the formation of the metastable T1 phase.

## 1. Introduction

Yttria-stabilized zirconia monolithic systems (ZrO_2_–Y_2_O_3_) for all-ceramic restorations were initially introduced to reduce the risk of chipping and delamination in zirconia-based crowns veneered with porcelain. This single-piece restoration design not only minimizes the risk of structural failure but also offers enhanced control over sintering processes, reducing pore formation that could lead to long-term degradation [1,2]. However, the transition to monolithic zirconia restorations presented a unique challenge to clinicians due to their limited optical adaptability. Soon after they came into widespread clinical use, the issue of natural appearance in monolithic zirconia restorations was posed [3]. In order to compensate for esthetic deficiency, various gradient technology (GT) procedures have been developed for the production of green zirconia components for simulating the shade gradient of human teeth.

Multichromatic zirconia [4] was implemented by combining layers with diverse pigments to achieve a gradual shade transition from the cervical to the incisal part of the restoration, mimicking natural tooth esthetics. The gradient color effect is created by adding a small amount of shading elements, like iron and rare-earth elements, to a white zirconia base. The only distinction among the layers is in the pigment composition, resulting in notable variations in shade while not affecting the translucency of the layers as anticipated [5]. Unlike conventional multilayer blocks, the multichromatic profile maintains consistent yttria concentrations across all layers. This uniform stabilizer oxide content also ensures similar flexural strength throughout the layers. Contrary to multichromatic multilayer zirconia, monochromatic multilayer zirconia is characterized by variation between tetragonal and cubic phase content, balancing translucency and strength to create a durable, visually pleasing, and harmonious material with a monolithic structure. This phase variation directly influences the material’s microstructure, which, in turn, plays an important role in determining the optical properties.

The typical microstructure of dental yttrium-stabilized tetragonal zirconia (Y-TZP) consists predominantly of the tetragonal phase (S.G. *P*4_2_/*nmc*), sintered to over 96% of its theoretical density [6]. The inclusion of alumina (Al_2_O_3_) in a small amount (at a concentration of 0.25 wt.%) in the starting mix of powders enhances the sinterability of doped zirconia, decreases porosity, and usually results in a reduced grain size (typically below 80 μm) in the obtained ceramic [7,8].

Due to the different structural and optical properties of tetragonal (S.G. *P*4_2_/*nmc*) and cubic (S.G. *Fm*-3*m*) phases of zirconia, the optical behavior of the final restorations will be significantly affected by their phase composition [9,10,11]. The cubic phase exhibits a more stable crystal structure with less strain inside. This stability is due to its symmetrical crystal structure, where all three axes are of equal length (*a* = *b* = *c*), forming a perfect cubic lattice [12]. The inherent symmetry and thermodynamic stability of the cubic phase of zirconia make it less susceptible to changes under external conditions. In contrast, the tetragonal phase, with its asymmetrical unit cell, is more prone to distortion, strain, and phase transformation with yttria substitution but also with changes in processing. At the same time, the crystallite size of the tetragonal phase can be changed more easily. The structural characteristics of these two phases determine the optical behavior of zirconia-based materials. The tetragonal zirconia gives rise to birefringence, causing high opacity, while the cubic phase of zirconia with larger grains (influenced by 25–50 wt.% of cubic phase) contributes to higher light transmission and transparency [13]. The phase transition from tetragonal to cubic phase is expected at yttrium concentrations of approximately 10%, when the cubic phase becomes more stable. A fully stabilized cubic phase can only be achieved at concentrations greater than 8%, while at lower concentrations, the tetragonal phase is much more stable since a lower dopant concentration favors the stabilization of the tetragonal phase [14].

Multichromatic zirconia restorations can be prepared for final sintering in two main ways: by shaping blocks that are already partly sintered or by using modern 3D printing techniques. The most common printing methods include SLA, DLP, robocasting, and material jetting [15]. Unlike milling, these techniques do not always require sintering during the fabrication step. This is important because the time and temperature of sintering have a significant effect on the internal structure of 3D-printed ceramics, while in pre-sintered blocks, sintering mainly serves to strengthen and stabilize the material.

An advantage of soft milling of pre-sintered blocks over 3D printing is that restorations fabricated from pre-sintered blocks exhibit greater structural reliability as 3D printing may show interlayer defects after sintering [16]. Such defects could reduce the optical quality of the restorations and limit their esthetic integration into natural teeth.

In subtractive manufacturing, dental restorations are usually produced by soft milling of partially sintered materials or by hard milling of fully sintered materials. Hard milling involves a longer processing time and frequent tool replacements. Due to the exceptional hardness and flexural strength of fully sintered monolithic zirconia, hard milling is primarily used for manufacturing zirconia abutments and implants [17]. Hard machining can create internal microcracks in the structure of fully sintered 3Y-TZP (yttria-stabilized tetragonal zirconia polycrystal) blocks, leading to an increase in the monoclinic phase (S.G. *P*2_1_/*c*) [18]. This phase transformation can result in surface microcracking, making the material more susceptible to low-temperature degradation (LTD). In contrast, soft milling of partially sintered restorations needs a shorter milling time (approximately 15 min), as opposed to hard milling, which requires a time at least three times longer [19]. Therefore, soft milling, as a cost-effective protocol, is expected to allow better control over the mechanical properties of the final restoration.

However, an ongoing issue with the soft milling procedure is that the additional sintering step is time-consuming. To make zirconia crowns available as chairside options, it became necessary to reevaluate sintering protocols. For that reason, a rapid sintering protocol was introduced and studied [20]. In addition to being time-effective, rapid sintering eliminates the need for temporization and reduces patient discomfort [21]. There has previously been considerable controversy around this concept. First, the clinical relevance was not fully understood because it was unclear how rapid sintering affected the microstructure, structural parameters, phase composition, and consequently, the optical properties of ceramic crowns. The optical properties of zirconia restorations are determined by the relative balance of tetragonal and cubic phases, which sintering parameters can alter. Rapid sintering, although clinically beneficial for chairside use, may compromise phase stability and lead to color deviations. Optimizing sintering protocols is essential to achieve reliable esthetic outcomes.

With growing interest from the scientific community in this complex problem, and with color stability emerging as a critical esthetic parameter, the consensus on visual assessment of ceramic crowns centers on two primary thresholds for evaluating color variances [22]. It is crucial to underline that the 50:50% thresholds (perceptibility and acceptability) are traditionally highlighted, with the acceptability threshold (AT) carrying greater significance than the perceptibility threshold (PT). Achieving a color match between a zirconia crown and natural teeth below the 50:50% perceptibility threshold denotes a near-perfect match, where 50% of lay observers can perceive a color difference, while the other 50% cannot. As far as acceptability, the value of 50:50% refers to the situation where 50% of lay observers are in agreement. From a CIELAB perspective, a ΔE (total color change) value of 1.2 corresponds to the 50:50% PT, whereas a ΔE value of 2.7 corresponds to the 50:50% acceptability threshold (AT) [22]. Color matches below the 50:50% PT are considered optimal. Yet, reaching such an imperceptible match can be challenging, expensive, and often unnecessary.

From a biological point of view, polishing, glazing, and pigment staining of zirconia can all yield surfaces that are highly biocompatible. The most important factors in biological response are surface smoothness and chemistry: a smoother surface (whether achieved by polishing or glazing [23]) promotes fibroblast adhesion, spreading, and proliferation, whereas rougher surfaces are less favorable. In vitro, gingival fibroblasts show attachment, high viability, and normal morphology on polished and glazed zirconia, even when pigments are present [23]. The goal is to form a well-finished zirconia (especially polished or appropriately glazed), which allows gingival tissues to integrate and maintain health, forming an epithelial seal and connective tissue adhesion with minimal inflammatory infiltrate.

By associating optical parameters with phase composition, this investigation clarifies the layer-specific sensitivity of multichromatic zirconia to a modified sintering protocol, as evidence in the existing literature remains scarce. The objective of this study was to determine the color change in a conventional and rapid-sintered multilayer zirconia. The research hypothesis was that there would not be differences between the optical parameters when comparing conventional and rapid-sintered zirconia. The additional aim of the study was to assess the cellular biocompatibility and adhesion of the polished and glazed surface of multichromatic zirconia.

## 2. Materials and Methods

### 2.1. Sample Preparation

**Figure 1:**
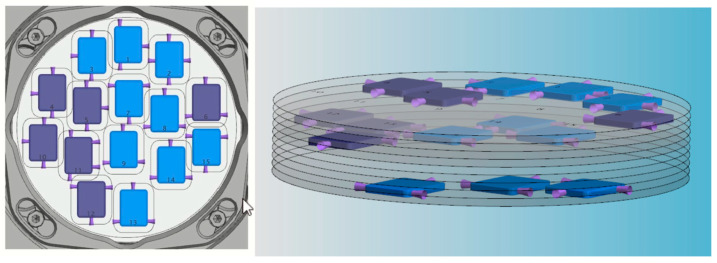
Sample nesting in CAM software.

The materials used in the present study were Multichromatic Katana STML (Kurary Noritake Dental, Tokyo, Japan) disks, A2 shade. Further samples were designed with CAD exocad DentalCAD software (v 2.3 Matera), followed by nesting in CAM Wieland Zenotec CAM software (v 2.0.049) to secure accurate positioning and precise milling (Figure 1). Pre-sintered disks were cut by a dry milling procedure, layer by layer (N = 4), as specified in the manufacturer’s instructions. Milling was performed using a dental milling machine, VHF-K5 (vhf, Ammerbuch, Germany). Milled samples were subjected to sintering following conventional (C) and rapid, i.e., speed (S) sintering methods, according to the manufacturer’s recommendation, using the furnace LHT 01/16 TURBO FIRE (Nabertherm, Lilienthal, Germany).

**Figure 2:**
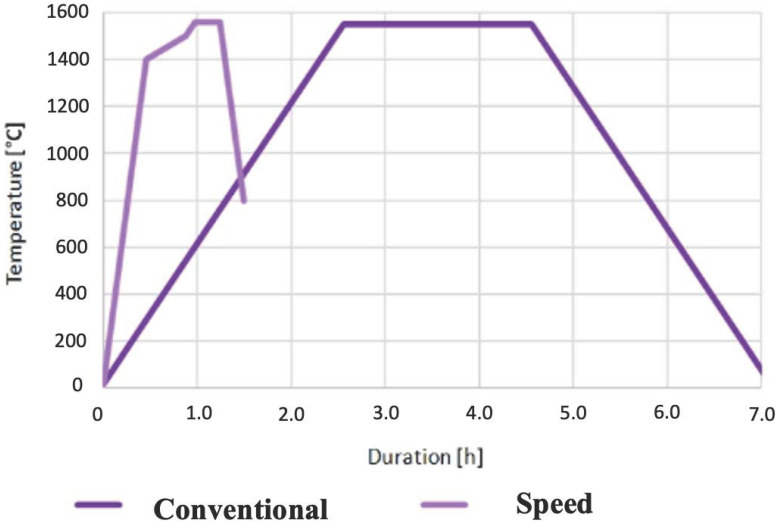
Sintering parameters for conventional and speed protocol.

The evolution of temperature over time during these two protocols, T(t), was monitored automatically (Figure 2). According to this value, the samples were separated into two groups:

(a) Control group (samples denoted as CS), obtained by conventional sintering with heating and cooling as follows:
  - Specimens were heated with a heating rate, ΔT/Δt = 10 °C/min up to T = 1550 °C, followed by holding for 2 h at that temperature, and then cooled for 153 min with a cooling rate of 10 °C/min to 20 °C before removing from the furnace. The total sintering time was 7 h.
(b) Experimental group (samples denoted as SS), obtained by speed sintering, were subjected to the followed protocols:
  - Specimens were heated with a heating rate 50 °C/min up to 1400 °C, then with 4 °C/min up to a temperature of 1500 °C, and then, for the last 16 min, with 10 °C/min up to 1560 °C. Cooling was carried out with a cooling rate of 50 °C/min down to 800 °C, dwelling for 5 min, when the specimens were removed from the furnace and left to cool down to room temperature (for approximately 15 min). The total sintering time was 90 min.

After sintering, the final dimensions of the samples were 10 × 13 × 1 mm. Additionally, the sintered samples were subjected to surface characterization treatment. Half of them were polished, and the other half were glazed.

(a) Polished specimens underwent dry polishing, performed with SagemaxNexxZr (Sagemax Bioceramics, Federal Way, WA, USA) Shine Kit diamond rubber polishers (rubber polishers for pre-polishing and high-gloss polish with diamond paste).
(b) Glazed specimens underwent transparent aluminosilicate glass deposition on a surface.

### 2.2. Color Measurements

Color measurements were performed using the digital reflectance spectrophotometer Datacolor SF300 (Datacolor, Rotkreuz, Switzerland) in the 400–700 nm range under the D65 standard illuminant using the 10° standard observer. For each specimen, measurements were conducted four times, and their average was recorded. On the basis of measured CIE color coordinates (lightness, L*; red–green value, a*; and yellow–blue value, b*), color difference (ΔE*) was determined as follows:

$$\Delta E*=\sqrt{{\left(\Delta a*\right)}^{2}+{\left(\Delta b*\right)}^{2}+{\left(\Delta L*\right)}^{2}}$$

where ΔL* is the color lightness difference between analyzed specimens, Δa* is the red/green difference between analyzed specimens, and Δb* is the yellow/blue difference between analyzed specimens [24].

### 2.3. X-Ray Diffraction (XRD)

The X-ray diffraction experiment was carried out on a Rigaku SmartLab diffractometer (Rigaku Co., Tokyo, Japan) equipped with Cu Kα,β sources of radiation (30 mA, 40 kV) and a Dtex 250 detector. XRD patterns of the samples were collected in a Bragg–Brentano geometry, in the range of 10–90° 2θ, with a step size 0.02° and a counting rate of 1°/min. The measurements were carried out on a surface of the sintered samples. To investigate the effect of the sintering protocol on a possible phase change in samples, the collected X-ray diffraction data were analyzed by the Rietveld method. Rietveld refinement was performed using integrated PDXL2 software incorporated into the SmartLab Studio package (Rigaku Co.). Peak profiles were fitted using the split pseudo-Voigt function. The tetragonal (S.G. *P*4_2_/*nmc*) and the cubic (S.G. *Fm*-3*m*) yttria-doped zirconia phases were introduced in the initial structural model applying Auto Search (suggested the ISCD cards with the best agreeing data). Microstructural analysis, based on the Halder–Wagner method [25], allowed us to estimate the values for crystallite size, *D*_xrd_, and lattice strain, ε.

### 2.4. Physical Analysis: Contact Angle Measurements

The wettability of the surfaces was assessed in controlled laboratory conditions (22.5 ± 0.2 °C). Each specimen was positioned on a measurement bench, after which droplets (2 µL) of distilled water, diiodomethane, and ethylene glycol were carefully dispensed onto the surface with a micropipette (Finnpipette, Thermo Fisher, Helsinki, Finland) at a vertical angle and 4 mm distance. Contact angle (θ) values were recorded 1 s after the liquid drop established contact with the sample. The experimental arrangement included a Canon 77D digital camera combined with a 100 mm ultrasonic macro lens (Canon, Tokyo, Japan) mounted on a fixed stand. The images obtained were processed using ImageJ software (Contact Angle plugin, version 1.5t, NIH/LOCI, University of Wisconsin, Madison, WI, USA). The surface free energy of polished and glazed specimens was subsequently determined according to the Owens–Wendt–Kaelble model with the aid of an online SEC calculator [26,27].

### 2.5. Biocompatibility Evaluation of Human Gingival Fibroblasts

Gingival tissue from two healthy donors was used for human gingival fibroblast (hGFs) isolation, after obtaining written consent. Gingival tissues were minced into approximately 1 mm^3^ fragments and subjected to the outgrowth method for cell cultivation. The minced tissue was transferred to 25 cm^2^ culture flasks containing complete growth medium (DMEM/F12 with 10% FBS and 1% antibiotics, sourced from Gibco, ThermoFisher, Waltham, MA, USA) and incubated at 37 °C in a humidified atmosphere with 5% CO_2_. The cells acquired following the third passage were utilized in the study.

Biocompatibility testing involved MTT and lactate dehydrogenase (LDH) assays. Zirconia disks (polished and glazed) were positioned in 24-well plates, with 2 × 10^4^ cells seeded onto each disk. The disks were incubated in freshly prepared growth medium at 37 °C in a humidified atmosphere containing 5% CO_2_ for 24 h. The medium was utilized for the LDH assay, conducted in accordance with the guidelines provided by the manufacturer, CyQUANT™ LDH Cytotoxicity Assay (Catalog no. C20300, ThermoFisher, Waltham, MA, USA). In the MTT assay, disks with attached cells were moved to a new 24-well plate, and 500 µL of a solution containing 3-(4,5-dimethylthiazol-2-yl)-2,5 diphenyltetrazolium bromide (MTT, 0.5 mg/mL) (Sigma-Aldrich, St. Louis, MO, USA) was added to each well, followed by a 4 h incubation. The supernatant was discarded, followed by the addition of 500 µL of dimethyl sulfoxide (Sigma-Aldrich, St. Louis, MO, USA) to each well. The plate was subsequently placed on a shaker for 20 min at 250 rpm, in the dark, at 37 °C. The colored solutions extracted from 24-well plates were transferred to a new 96-well plate. Optical density was assessed at 570 nm utilizing a microplate reader RT-2100c (Rayto, Shenzhen, China). The percentage of cell viability was determined by comparing the experimental group to the control group (cells seeded on plastic) using the following formula: (OD (sample) − OD (Blank))/(OD (control) − OD (Blank)) × 100. The experiment was conducted in three iterations.

A cell adhesion assay was performed to assess the potential for cell attachment to materials. Cells (1 × 10^4^) were seeded onto materials and cultured in an incubator with 5% CO_2_ at 37 °C for 24 h. Following the incubation period, non-adherent cells were quantified utilizing a hemocytometer. As a control, an equivalent number of cells were seeded in unoccupied 24-well culture plates. The adhesion rate (%) is calculated using the formula (number of seeded cells − non-adhered cells)/(number of seeded cells) × 100. This formula [28] was employed to determine the number of non-adherent cells following a 24 h treatment. The experiment was conducted in three iterations.

## 3. Results

### 3.1. Optical Properties

The mean values of CIELAB color coordinates after different sintering protocols are given in Table 1.

#### 3.1.1. Differences in Total Color Change in Speed-Sintered Samples

Table 1 shows the color change, E*, of speed-sintered (SS) Katana specimens, which were polished or glazed after sintering. The polished Katana specimens showed the highest change in color. ∆E* values of samples in all layers were above the perceptibility threshold (PT) (∆E* > 1.2). The value detected only in the enamel layer (EL) was higher than clinically acceptable (∆E* > 2.7). It was also observed that the EL of speed-sintered and polished Katana specimens became reddish (a*+) and yellowish (b*+). At the same time, ∆E change was highly influenced by ∆b* within all layers of Katana specimens.

#### 3.1.2. Differences in L* and C* Values

For polished Katana samples, the enamel layer (EL) exhibited the highest color change and lightness difference (∆L) and contributed the most to color change in this sample. This value slightly exceeded the AT for lightness difference (∆L* > 2.44) [29]. Upon evaluating changes in lightness, all polished specimens showed a decrease in ∆L from the enamel layer (EL) to the body layer (BL). Similar results were observed for glazed samples.

Regarding changes in chroma, none of the tested samples exhibited values higher than the AT ones (∆C* < 3.15) [29]. Nevertheless, it should be noted that the change in chroma was the main factor influencing the color variation in the BL of glazed Katana samples.

### 3.2. The Crystal Structure of Sintered Katana STML Samples

**Figure 3:**
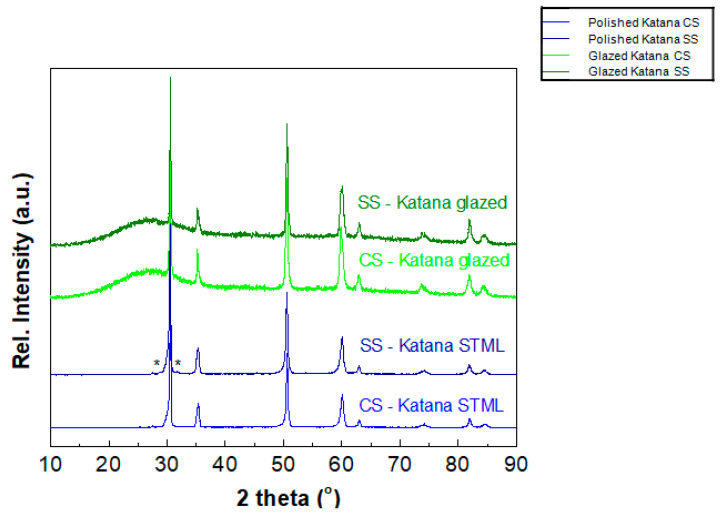
XRD data for glazed and polished EL of conventionally sintered, CS-, and speed-sintered, SS-, Katana STML. Asterisks (*) indicate a presence of monoclinic crystal structure (S.G. *P*2_1_/*c*) in yttria-stabilized zirconia.

**Figure 4:**
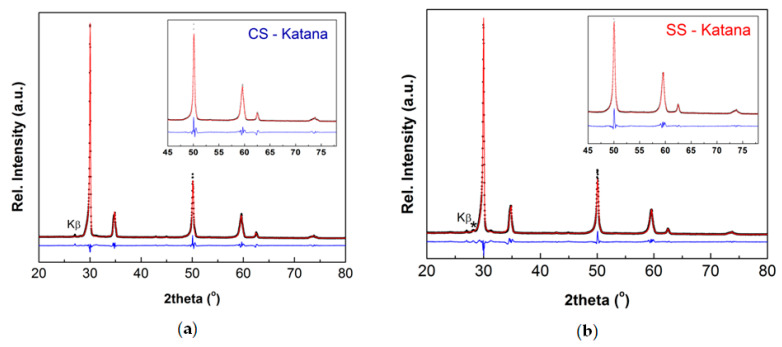
Refined XRD data of (**a**) conventionally sintered (CS-) and (**b**) speed-sintered (SS-) Katana STML. Asterisks (*) indicate a presence of monoclinic phase (S.G. *P*2_1_/*c*).

Experimentally collected XRD data for polished and glazed Katana STML samples, sintered following the conventional protocol (CS) or speed sintering route (SS), are shown in Figure 3. Given the fact that only EL of Katana polished samples showed changes in color parameters beyond AT values, a subsequent analysis of their phase structure was conducted. Two low-intensity peaks labeled by asterisks in the sample SS-Katana STML indicate a small amount of monoclinic phase (S.G. *P*2_1_/*c*). The crystal structure refinement of the conventionally, CS-, and speed-sintered, SS-, Katana, along with the phase quantification, were performed using the obtained XRD data and PDXL2 software. Refinement was performed by testing different structural models and keeping the stoichiometry of the samples at Zr_0.935_Y_0.065_O_0.984_. Among the most plausible approaches, we singled out the model including the presence of two phases for the CS-Katana STML and three phases for the SS-Katana STML. The model for the CS-Katana in which two tetragonal phases coexisted was more competitive compared to the model with one tetragonal (S.G. *P*4_2_/*nmc*) and one cubic (S.G. *Fm*-3*m*) phase. Since better agreement was achieved for the CS-Katana using the second model, the results of refinement using one tetragonal (T) and one cubic (C) phase are given below. For the SS-Katana sample, an additional tetragonal phase (T1) was introduced during refinement. Figure 4 shows the results of Rietveld refinement for the CS- and SS-Katana samples. Table 2 list the structural parameters, as well as the crystallite size, D_XRD_, and lattice strain, ε, estimated by the Halder–Wagner method [25]. The results of the quantitative analysis (i.e., estimated phase composition) based on the RIR method (weight %) are also given in Table 2.

### 3.3. Contact Angle Measurements

The water wetting angle measurements of polished and glazed zirconia samples showed that both surfaces presented hydrophilic behavior (Table 3). Still, for glazed zirconia, lower contact angle values were observed in comparison to polished zirconia.

### 3.4. Comparable Cell Viability and Attachment on Polished and Glazed Zirconia Surfaces

**Figure 5:**
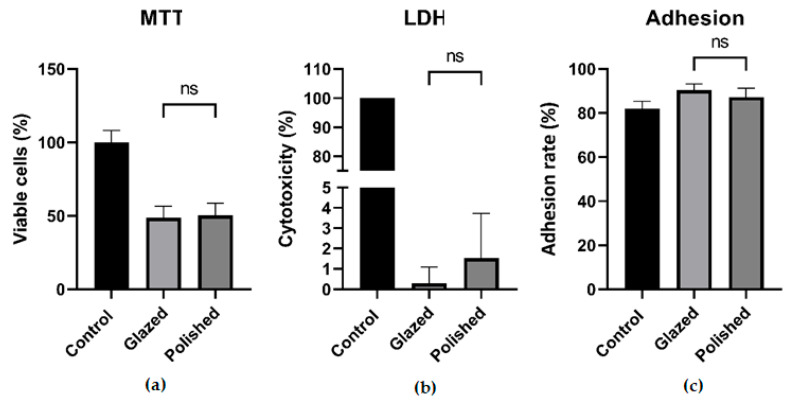
MTT (**a**), LDH (**b**), and cell adhesion (**c**) assays of hGFs seeded onto polished and glazed zirconia disks; ns—not significant.

After 24 h of cell exposure to the polished and glazed zirconia, mitochondrial activity and LDH levels, which correspond to cell viability, were similar (*p >* 0.05). The cell attachment exhibited comparable results for both polished and glazed materials (*p* > 0.05) (Figure 5).

## 4. Discussion

This study verified the color stability of each layer of multichromatic blocks independently. The results obtained offered partial evidence against the research hypothesis, indicating that the speed sintering protocol has an effect on the optical properties of Katana zirconia, but only in the enamel layer.

The currently available literature regarding changes in optical parameters in rapid-sintered ceramics is mainly focused on research concerning changes in translucency [21,30,31,32]. Studies revealed that sintering speed did not affect the translucency of Katana STML zirconia [32]. According to recent research, speed-sintered zirconia can show a slight increase in translucency and lightness, but these changes may not be perceptible to the human eye in a clinical context. These findings suggest that the optical adjustments achieved are often subtle and primarily noted in specific layers of multilayered zirconia rather than uniformly across the material. Our findings suggest that these changes are only notable in the enamel layer in Katana.

The obtained results were subsequently compared to the clinical threshold values previously reported in clinical research [33]. As per literature data, the perceptibility threshold was established at 1.2, while the threshold for acceptability was set to 2.7 [29]. This comparison was essential to contextualize our findings within clinically relevant parameters. The range of ΔE of the polished Katana specimens was between 1.5 and 3.0, meaning that obtained values were higher than ΔE PT (>1.2) in the case of all layers. However, in terms of the acceptability threshold, only the enamel layer exhibited values exceeding the ΔE AT (>2.7). This finding is of particular relevance since the enamel layer represents the most esthetically critical zone of multichromatic zirconia. This result therefore indicates that speed sintering may induce a clinically perceptible color mismatch, specifically in the enamel portion of the future restoration, which is required to mimic the surrounding natural dentition. This directly relates to our objective of evaluating the optical stability of individual layers in order to ensure predictable esthetic outcomes.

Analyzing the results in the group of glazed samples from the perspective of PT, the results were similar to the polished samples, as ΔE values ranged between 1.5 and 2.5 and thus were higher than ΔE PT in all layers. A color difference was not observed in the mean of the acceptability threshold, as all the results were lower than 2.7.

The results also show that the polished samples exhibited a more significant change in lightness compared to the glazed samples. This outcome is expected, as studies [34,35] examining the difference in lightness between polished and glazed crowns agree that, under the same conditions, polished crowns appear brighter. This is because the polishing process creates smooth, glossy surfaces that enhance specular light reflection. Additionally, it has been reported that diamond abrasive pastes used for polishing can impart a luster to the surface. Glazing creates a surface texture with dots and ridges that represents areas of highest convexity, where light is more likely to reflect. Conversely, pits and concavities on the surface are areas where light rays are more likely to be absorbed. Clinically, this suggests that the choice between polishing and glazing directly affects the perceived brightness of the final restoration.

Taking into account phase quantification of Katana STML samples, literature data suggest variations in the phase composition depending on the sintering protocol. Also, the phase composition depends on the mol.% of Y_2_O_3_ that stabilizes tetragonal zirconia polycrystal. According to some authors, the cubic phase in Katana STML can range from 55 wt.% to 70 wt.% [36]. We are aware that the XRD technique is not sensitive enough to detect the cubic–tetragonal phase transition in zirconia ceramics, primarily because oxygen has a relatively low atomic scattering factor. Similarly, electron diffraction and Raman scattering are not effective in measuring oxygen displacement, which is a key factor in quantitatively analyzing the cubic–tetragonal phase transition using an order parameter [37,38,39]. Here, additional difficulty arises from the nanoparticulate nature of the CS- and SS-Katana samples (visible through broadened reflections). According to the phase diagram of zirconia [40], phase transition from tetragonal to cubic phase can occur due to increased yttria content at around 10% or an increased cooling rate. The results of our study, determined based on fixed occupancy at Zr_0.935_Y_0.065_O_0.984_ and presented in Table 2, reveal that speed sintering caused a decrease in the cubic (C) phase and an increase in the total amount of tetragonal (T_tot_) phases. Additionally, a small amount of the monoclinic (M) phase is noticed (Figure 2). According to the literature data [41], two possible factors can explain the presence of M-phase. Primarily, higher heating rates may not provide sufficient time for the complete transformation of the monoclinic to tetragonal phase, resulting in residual monoclinic phase. Secondarily, the effect of higher cooling rates may compromise the stability of the tetragonal phase, potentially leading to unwanted monoclinic transformation. From a clinical point of view, this can increase susceptibility to low-temperature degradation (LTD), compromising the longevity of the restoration.

Correspondingly, in a study where Katana STML was sintered at 1560 °C for a holding time of 7 min, a decrease in cubic phase was noted, together with an increase in tetragonal phase, with elevated tetragonality [42]. Reports from other studies also indicate that when dental zirconia with less than 7.5 mol% yttria is rapidly cooled from temperatures above 1425 °C, it may form a metastable tetragonal phase instead of the cubic phase [43]. In our study, when both the heating and cooling rates were five times higher for the speed-sintered samples compared to the conventional ones, the appearance of a metastable tetragonal phase (T1) was also noticed. In addition, the heating rate during sintering changed the lattice parameters of all phases (see Table 2). The tetragonality value, c/$\sqrt{2}$a, of the T1-phase is higher than 1, indicating that this phase is in the lower yttrium region [44]. This can be due to the difficulty in reaching the equilibrium state during speed sintering. The rapid thermal expansion causing internal thermal stress does not allow enough time for the grain boundaries to equilibrate. The low yttrium region can be selectively transformed into monoclinic (M) phase. Indeed, two peaks labeled by asterisks in Figure 2 are linked to the M-phase. At the same time, by increasing the heating and cooling rate, ΔT/Δt, during the sintering process, the crystallite size, *D*_xrd_, stayed almost unchanged, while the lattice strain, ε, increased in the speed-sintered sample (SS- Katana STML) (here, ε mostly arose from dislocations inside the crystal structure) [37]. The phase fraction (weight%) of cubic (C-) and tetragonal (T-) phases, calculated by the RIR method, is given in Table 2, too.

It is important to identify the difference between the metastable, transformable tetragonal phase and the non-transformable tetragonal phase, since only the former can undergo the T–M transformation that strongly affects mechanical behavior [45].

In this study, the focus was on optical properties; however, the literature indicates that the presence of T1 also influences mechanical performance. Because XRD alone cannot differentiate between the transformable and non-transformable tetragonal fractions, the quantitative contribution of each remains unclear. Therefore, further investigations are needed to confirm the potential of the T1 phase obtained by rapid sintering to undergo transformation, as observed in transformation-toughened zirconia with martensitic effects. Additional techniques beyond XRD are required to provide reliable evidence of the phase fractions. Finally, it cannot be assumed that the material necessarily contains a higher proportion of non-transformable tetragonal phase solely on the basis of yttria concentration.

Since the available literature-based evidence suggests a wide variation in sintering protocols (different furnaces, heating rates, and dwell times), the rational comparability of results remains challenging, emphasizing the importance of standardization. Excessively high heating rates should be avoided, as excessively rapid heating may lead to inhomogeneous densification and defects within the layers of multichromatic zirconia. Similarly, dwell times at peak temperatures should be carefully shortened, since prolonged exposure to high temperatures can promote excessive grain growth and reduce translucency, thereby altering the overall optical behavior. Regarding the cooling stage, a two-step protocol is preferable, as gradual cooling reduces residual stresses and helps preserve both esthetic and phase fraction properties in multichromatic zirconia blocks.

The fact that polycrystalline zirconia contains a predominantly crystal structure characterized by smaller grains and more boundaries per surface compared to glass–ceramic materials favors the possibility of final polishing instead of only glazing. The influence of different surface finishes on bacterial adhesion and colonization is well documented. Materials with surface ability to bind water molecules can influence various processes, including enhanced microbial attachment to their surface. As reported in previous research [46], hydrophilic surfaces may also help to reduce antagonist wear. Improved lubrication during contact reduces friction and wear between antagonistic surfaces.

However, the literature lacks evidence about differences in cellular adhesion on polished versus glazed zirconia. In the gingival area, the surface wettability of ZrO_2_ plays an important role in its soft-tissue integration via modulation protein adsorption and properties of hGF [47]. By tuning zirconia (via polishing, glazing, etc.), its hydrophilicity could be changed, influencing the hGF response [47,48]. The data suggest that the most favorable contact angle value for cell adhesion and protein adsorption is around 40, which characterizes moderate hydrophilicity [49,50]. Generally, smoother and more hydrophilic zirconia surfaces promote superior fibroblast adhesion and proliferation. In a recent study, an investigation of polished versus glazed zirconia yielded comparable cell outcomes—both finishes were smooth enough that their minor wettability differences did not appreciably alter HGF adhesion/proliferation [51]. This is in line with our study, as significant differences in cellular adhesion and viability were not detected. Although the polished and glazed surfaces did not show differences in promoting fibroblast viability and attachment, further research is needed to better understand this interaction and confirm the results. There is significant room for further investigation into, and optimization of, the surface characteristics of ZrO_2_ ceramics to enhance biological interactions with hGFs.

Based on current scientific evidence, polished zirconia restorations demonstrate superior long-term performance compared to glazed zirconia [52,53]. Polished surfaces exhibit enhanced wear resistance, maintain surface integrity over time, and cause less abrasion to the opposing dentition. In contrast, glazed layers tend to degrade under functional loading, leading to a loss of gloss, increased roughness, and compromised esthetics. From a biological standpoint, smoother, polished surfaces provide more favorable conditions for soft tissue adhesion and long-term stability at the restoration–gingiva interface. Therefore, considering both longevity and biofunctionality, polishing appears to be a more reliable finishing method than glazing for zirconia restorations.

Nonetheless, our study has several limitations. Research comprised an in vitro investigation conducted on a single cell type, specifically, human gingival fibroblasts, under short-term culture conditions. The in vivo biological response, characterized by intricate interactions among epithelial cells, immune cells, and osteoblasts, may not align with our findings.

## 5. Conclusions

In this work, multichromatic Katana STML zirconia was rapid-sintered using a protocol that included heating rates incorporated into three steps, 50 °C/min to 1400 °C; 4 °C/min to 1500 °C; 10 °C/min to 1560 °C (16 min), and subsequent cooling rates incorporated into two steps: 50 °C/min to 800 °C (5 min dwell), and removal from the furnace to room temperature (~15 min).

The sample color measurements in this study showed that the rapid-sintered multichromatic Katana STML had higher lightness compared to the conventionally sintered sample in the area corresponding to the incisal part of the crown. All layers demonstrated ΔE values above the perceptibility threshold (ΔE > 1.2), while only the polished enamel layer (ΔE* = 3.01) exceeded the acceptability threshold (ΔE* > 2.7), resulting in a clinically perceptible mismatch in the optically dominant zone of the final restoration.

XRD analysis of multichromatic zirconia revealed the appearance of an yttria-lean T1-phase with high tetragonality in the speed-sintered zirconia samples. Rapid sintering influences the microstructure of zirconia ceramics by promoting the formation of the T1 phase. This occurs due to the shortened time for atomic diffusion within the crystal lattice, which prevents the complete development of the cubic phase and instead results in a tetragonal phase close to cubic symmetry.

Glazing as a surface finishing procedure increased hydrophilicity and surface energy values. Both polished and glazed zirconia demonstrated comparable cell viability and attachment.

Consequently, the properties of dental zirconia achieved via rapid sintering were not identical to those of zirconia processed using traditional sintering protocols. In conclusion, the changes in the sintering protocol could sacrifice the total color change in the material and thus compromise the esthetic appearance of the final restoration.

## Figures and Tables

**Table 1:** Color coordinates for SS Katana specimens.

	Layer	ΔE*	ΔL*	ΔC*	ΔH*	Δa*	Δb*
Polished	Enamel	3.013	2.453	1.621	−0.659	0.444	1.692
	Transition 1	1.643	0.511	1.544	−0.227	0.095	1.558
	Transition 2	1.514	0.362	1.482	−0.298	0.245	1.446
	Body	1.496	0.600	1.337	−0.298	0.326	1.331
Glazed	Enamel	2.580	1.593	1.888	−0.747	0.345	2.000
	Transition 1	1.528	0.168	1.466	−0.398	0.196	1.506
	Transition 2	1.925	−0.247	1.683	−0.488	0.263	1.832
	Body	2.196	−0.925	2.256	−0.274	0.244	2.259

**Table 2:** The results of Rietveld refinement for the EL of CS- and SS-Katana samples.

	Heating Rate (°C/min)	Phase	Lattice Parameters (Å)	Phase Fraction (wt.%)	Tetragonality, $c/\sqrt{2}$*a*	Crystallite Size, *D*_XRD_ (nm)	Lattice Strain, ε (%)
**CS-Katana**	10	Tetragonal (S.G. *P*4_2_/*nmc*)	*a* = *b* = 3.633 (7) *c* = 5.124 (8)	54 (8)	0.997	17 ± 2	0
		Cubic (S.G. *Fm*-3*m*)	*a* = *b* = *c* = 5.137 (7)	46 (8)			
**SS-Katana**	50	Tetragonal (T)	*a* = *b* = 3.627 (7) *c* = 5.157 (8)	49 (3)	1.005	19 ± 2	0.2 (2)
		Tetragonal (T1)	*a* = *b* = 3.615 (7) *c* = 5.168 (8)	27 (2)	1.011		
		Cubic (C)	*a* = *b* = *c* = 5.189 (9)	24 (4)			

**Table 3:** Descriptive statistics of contact angle measurements (mean ± St. dev).

Liquid	Sample	
	Polished	Glazed
Water	45.1 ± 1.2	32.3 ± 1.8
Ethylene glycol	50.1 ± 3.1	28.1 ± 1.4
Diiodmethane	42.6 ± 2.0	35.5 ± 2.2

The calculated surface energy value for glazed samples was 67.2 mJ/m^2^, and for polished samples, it was 59.4 mJ/m^2^.

## Data Availability

The original contributions presented in this study are included in the article. Further inquiries can be directed to the corresponding author.
